# MRI Brain Classification Using the Quantum Entropy LBP and Deep-Learning-Based Features

**DOI:** 10.3390/e22091033

**Published:** 2020-09-15

**Authors:** Ali M. Hasan, Hamid A. Jalab, Rabha W. Ibrahim, Farid Meziane, Ala’a R. AL-Shamasneh, Suzan J. Obaiys

**Affiliations:** 1 College of Medicine, Al-Nahrain University, Baghdad 10001, Iraq; a.hasan4@colmed-alnahrain.edu.iq; 2 Faculty of Computer Science and Information Technology, University of Malaya, Kuala Lumpur 50603, Malaysia; hamidjalab@um.edu.my (H.A.J.); shamasneh@siswa.um.edu.my (A.R.A.-S.); 3 Informetrics Research Group, Ton Duc Thang University, Ho Chi Minh City 758307, Vietnam; 4 Faculty of Mathematics and Statistics, Ton Duc Thang University, Ho Chi Minh City 758307, Vietnam; 5 Data science Research Centre, University of Derby, Kedleston Rd, Derby DE22 1GB, UK; f.meziane@salford.ac.uk; 6 School of Mathematical & Computer Sciences, Heriot-Watt University Malaysia, Putrajaya 62200, Malaysia; s.obaiys@hw.ac.uk

**Keywords:** quantum calculus, fractional calculus, quantum entropy, deep learning, MRI classification

## Abstract

Brain tumor detection at early stages can increase the chances of the patient’s recovery after treatment. In the last decade, we have noticed a substantial development in the medical imaging technologies, and they are now becoming an integral part in the diagnosis and treatment processes. In this study, we generalize the concept of entropy difference defined in terms of Marsaglia formula (usually used to describe two different figures, statues, etc.) by using the quantum calculus. Then we employ the result to extend the local binary patterns (LBP) to get the quantum entropy LBP (QELBP). The proposed study consists of two approaches of features extractions of MRI brain scans, namely, the QELBP and the deep learning DL features. The classification of MRI brain scan is improved by exploiting the excellent performance of the QELBP–DL feature extraction of the brain in MRI brain scans. The combining all of the extracted features increase the classification accuracy of long short-term memory network when using it as the brain tumor classifier. The maximum accuracy achieved for classifying a dataset comprising 154 MRI brain scan is 98.80%. The experimental results demonstrate that combining the extracted features improves the performance of MRI brain tumor classification.

## 1. Introduction

Brain tumors are considered as a one of the deadliest diseases worldwide and impact the patients’ lives physically, cognitively and psychologically. Approximately, over 200,000 people suffer from these pathologies every year in the United States [1]. Most of these pathologies start elsewhere in the body and then spread to the brain. In normal tissues, the rate of growing and dying is kept under control. Uncontrolled growth rate leads to the creation of pathologic cells known as cancerous cells. Brain tumors incorporate heterogeneous cells with uncontrolled proliferation rates and differ significantly by their morphologic characteristics and genetic variations. The choice of treatment is determined by the location of the tumor, the cell type and the possible existence of other illnesses.

The most of the medical institutions use classification systems to recognize more than 120 types of brain tumors. Medical imaging technologies include many types, and the most common is the magnetic resonance imaging (MRI). Often, these technologies are used in combination to provide early diagnosis and to improve treatment possibilities [2,3].

MRI is considered as the standard noninvasive imaging technique. This technology uses radio-frequency signal and large magnetic field to excite tissues, then observe the behavior of proton’s orientation to produce a high resolution cross-sectional visualization of the inside of the body. The provided MRI scans include valuable detailed information to differentiate soft tissues with a high spatial resolution that may be up to 1 × 1 × 1-mm voxel size [4]. There are many different MRI sequences, all of them attempt to optimize the tissue contrast. This allows the measurement of the alterations in the tissue fluid and the production of different contrast visualizations. The standard sequences of MRI are: noncontrast T1-weighted (T1-w) which is used routinely to evaluate normal anatomy due to its high resolution and less artifact characterization; and T2-weighted (T2-w) which is an important sequence that is very suitable to detect pathologies, where they appear as a highly intense signal due to the high fluid content. Therefore, it is commonly used for the initial tumor assessment and for distinguishing tumor/non-tumor tissues [5]. Clinically, both T1-w and T2-w sequences are significantly relied upon in the diagnosing process, although some difficulties can be encountered when using these two sequences to discriminate pathologic from anatomic areas. This is particularly evident when the distribution of the voxel intensities of cerebral-spinal fluid, the gray matter of the brain and tumors appear close to each other [6]. Subsequently, a ferromagnetic medium (gadolinium) is generally combined with T1-w sequence to clarify the tumor boundaries. This sequence is named T1-weighted with contrast enhancement (T1C-w). The fluid-attenuated inversion recovery (FLAIR) sequences are reflecting the non-enhanced pathologic tissues [3]. The diagnostic process of medical images that is run by a group of clinicians, is considered as a subjective quality assessment. The diagnostic imaging techniques have been developed for the medical image quality assessment to add more objectivity to the subjective diagnosis as well as to reduce the erroneous diagnostic interpretation and helping clinicians to fast examining the massively increased number of MRI slices. These techniques are based on the characterization of tissues in MR images through texture analysis. The texture analysis is an efficient way to extract high-level information. Moreover, texture analysis was shown to discriminate between anatomic and pathologic tissues better than the human visual system.

In this study, we exploit the quantum entropy-LBP (QELBP) for feature extraction from the MRI brain scan with the deep-learning features (DL) that are extracted by the proposed convolutional neural networks (CNN). The DL feature extraction method able to extract more diverse features from each MRI brain scan. The QELBP and DL features are combined to improve the brain tumor classifier. The long short-term memory (LSTM) network is an addition category of the artificial recurrent NNs. LSTM can be applied to a variety of deep learning tasks. In this study, the extracted features are used as inputs to the LSTM that is then trained to classify the MRI brain into normal and abnormal scans. The rest of the paper is organized as follows: some recent related works are reviewed in Section 2. In Section 3, we present the full description of the proposed model. The detailed experimental consequences are investigated and discussed in Section 4, and lastly, Section 5 indicates the conclusions of the study.

## 2. Related Work

The automated systems for MRI brain scan classification remains challenging because of the variability and complexity of brain tumors. There are several studies that used texture features in the MRI brain classification and sometime many feature extraction techniques are combined to improve the classification accuracy [4]. Sachdeva et al. [7] proposed a system for assisting radiologists to classify MRI brain scans automatically. Multi texture features were used and combined with genetic algorithm (GA). Two classifiers; SVM and multilayer perceptron (MLP) were applied individually, these two classification techniques were compared and MLP was shown to be better than SVM. Nabizadeh and Kubat [5] proposed a fully automated system to classify the MRI brain scans. Their system was based on combining many standard feature extraction techniques, which were refined by using the principle component analysis (PCA). The achieved accuracy was 97.4% by using SVM to classify a dataset that included 25 MRI scans.

Texture features constitute an important aspect in MRI brain classification. They are widely accepted when combined with deep features which represent high level spatial features to provide significant advantages. Thus, they have started to outweigh the performance of the proposed models in various applications. Several studies have been proposed in this direction. Hasan et al. [8] proposed an automated system for classifying MRI brain scans into pathologic and normal MRI brain scans. The proposed system combined the features that are extracted by CNNs and the modified GLCM. The authors proved that the combination of the extracted features makes the classification accuracy significantly higher.

Recently, CNN has become increasingly prevalent in the field of machine-learning and feature extraction. Their success comes from increasing the number of layers, rectified linear units, regularization rules and effective use of data augmentation [9]. Chen et al. [10] proposed an approach for hyperspectral image classification using regularized deep feature extraction method. Experimentally, three conventional layers of CNN with a kernel size, of 4 and 5 with a pooling kernel of 2 in each layer were used. Liang and Li [6] applied the sparse representation of deep learning features for remotely sensed image classification. CNN was used to extract deep features, which represent high-level spatial features of the images. Finally, the support vector machine (SVM) was used to classify the deep extracted features. Lai and Deng [11] proposed a new medical image classification algorithm based on the combination of high level deep features and some selected texture features.

From the review of the MRI brain classification methods above, it can be observed that the most of the used CNN models are based on deep feature extraction, which works well with certain selected types of images. The proposed feature extraction of both the QELBP and DL models for automated MRI brain tumor classification are applied to enhance the accuracy of the diagnosis procedure. To summarize, the following are the major contributions of this study:

1. The proposed QELBP features as a texture descriptor;
2. The DL features as a deep feature extractor;
3. The 154 MRI brain scans which are collected from Al-Kadhimiya Medical City, Iraq.

## 3. Proposed QELBP–DL Model

**Figure 1:**
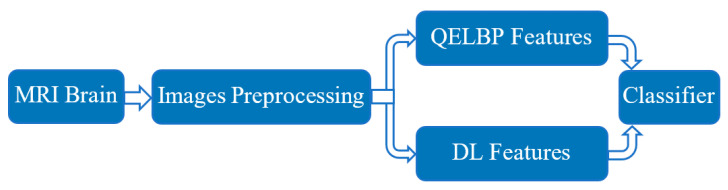
Proposed quantum entropy local binary patterns (QELBP)–deep-learning (DL) model.

The proposed method includes four main stages; MRI brain scans preprocessing, QELBP features extraction, DL feature extraction and finally MRI brain scans classification by using the LSTM network. The proposed model is shown in Figure 1.

### 3.1. Data Collection

In this study, a brain MRI scans dataset of 154 images of 512 × 512 pixels was collected from Al-Kadhimiya Medical City, Iraq. These comprise 77 scans of healthy people, 77 scans of patients suffering from different types of brain tumors. These data set was acquired by SIMENS and PHILIPS scanners. The former has a voxel resolution of (1 × 1 × 5 mm^3^), while the latter is (1 × 1 × 3 mm^3^). All of the collected scans were classified into normal cases or abnormal cases by the clinicians and a formal agreement was gained from the patients allowing using their MRI scans in this study. All subjects gave their informed consent for inclusion before they participated in the study. The study was conducted in accordance with the declaration of Helsinki and was approved by research, innovation and academic engagement ethical approval panel, University of Salford (Approval No.: CST15/54).

### 3.2. MRI Brain Scan Preprocessing

Prior to analyzing the MRI brain scans statistics, a set of popular preprocessing methods commonly used to reduce the effects of random fluctuations in the intensity distribution of MRI scans that may come from image noise, bias field effect, patient motion and respiration are used [12]. Therefore, several preprocessing methods are often implemented in the preparation of MRI scans. The MRI scan normally includes significant intensity variations, therefore, it is important to exclude these variations form any postprocessing steps [13]. The MRI scans were enhanced by Gaussian filter and normalized by the histogram normalization. To enable the use of all MRI scans from different scanners without bias, zero padding is used to adjust the dimensions of MRI slices to 512 × 512 pixels in resolution [14].

### 3.3. QELBP Feature Extraction

The main advantages of LBP are the simplicity and invariance to constant variations of image intensity. This characteristic makes LBP to be a good texture descriptor. Mathematically, basic LBP can be described by Equation (1) (see [15]).

(1) $$LBP_{p,r}\left(x_{k},y_{k}\right)=\overset{p-1}{\underset{k=0}{\sum }}f\left(g_{k}-g_{c}\right)2^{k},$$

where $g_{c}$ is the central pixel intensity value and $g_{k}$ represents the kth neighborhood pixel intensity value in the circular region (*p*, *r*), and *f* (*d*) is the thresholding function which is given by:

(2) $$f\left(d\right)=\{\;\begin{array}{c} 1,\;d\geq 0\\ 0,\;d<0 \end{array}$$

where *p* is the number of sampling points, and *r* the radius of the circle.

The image texture is characterized by the spatial distribution of image intensity values in a neighborhood. Moreover, the texture features include point operations, where each pixel is modified according to particular equation. This motivate us to apply quantum calculus (QC) for providing better texture feature enhancement. Inspired by the quantum, we propose the QELBP as a new feature extraction for MRI brain scans. QC is matching to customary normal calculus without the using of limits. QC denotes by “q-calculus” and formulates by [16]:

(3) $$\partial_{q}\;ℵ\;\left(\chi \right)=\;\frac{ℵ\left(q\chi \right)-ℵ\left(\chi \right)}{\left(q-1\right)\chi },$$

where ℵ indicates a function and χ designates the variable. One of the important function in QC is the natural logarithmic function, which is constructed by the formal [16]:

(4) $$ln_{q}\;\left(\chi \right)=\{\begin{array}{c} \ln\left(\chi \right)\;\;\;\;\;\;\;\;\;\;\;\;\;\;\;\;\;\;\;\;\;q=1,\chi >0\\ \frac{\chi^{1-q}-1}{1-q}\;\;\;\;\;\;\;\;\;\;\;\;\;\;\;\;\;\;\;\;\;q\neq 1,\;\chi >0\\ not-valid\;\;\;\;\;\;\;\;\;\;\;\;\;\;\;\;\;\;\;\;\;\chi <0 \end{array}.$$

The Marsaglia technique [17] is a pseudorandom digital sampling technique for creating a pair of independent random variables. The modified Marsaglia formula (M) is defined by the following formula:

(5) $$M\left(\chi ,\Upsilon \right):=\left(\frac{\chi }{\sqrt{\delta }}\;\sqrt{-2ln\;\left(\chi \right)},\;\frac{\Upsilon }{\sqrt{\delta }}\;\sqrt{-2ln\;\left(\Upsilon \right)}\right),$$

where *δ* is a unit circle given by $0<\delta :=\;\chi^{2}+\Upsilon^{2}<1.$ In our application, we assume *δ* = 1.

By substituting Equation (4) in Equation (5), we have the q-Marsaglia formula as follows:

(6) $$M_{q}\left(\chi ,\Upsilon \right):=\left(\frac{\chi }{\sqrt{\delta }}\;\sqrt{-2ln_{q}\;\left(\chi \right)},\;\frac{\Upsilon }{\sqrt{\delta }}\;\sqrt{-2\;ln_{q}\;\left(\Upsilon \right)}\right)$$

The QELBP is defined by employing (6).

We define the entropy change (Entropy difference) by using *g_k_* and *g_c_* combining

Marsaglia formula as follows:

(7) $$\Delta \Xi =\left(\frac{g_{k}}{\sqrt{\delta }}\;\sqrt{-2ln\;\left(g_{k}\right)}\right)-\left(\frac{g_{c}}{\sqrt{\delta }}\;\sqrt{-2ln\;\left(g_{c}\right)}\right)$$

Consequently, the q-difference entropy becomes:

(8) $${\left(\Delta \Xi \right)}_{q}=\left(\frac{g_{k}}{\sqrt{\delta }}\;\sqrt{-2ln_{q}\;\left(\;g_{k}\right)}\right)-\left(\frac{g_{c}}{\sqrt{\delta }}\;\sqrt{-2ln_{q}\;\left(g_{c}\right)}\right)$$

By substituting (8) in (1), we have the QELBP as follows:

(9) $$\begin{array}{c} QELBP_{p,r}\left(x_{k},y_{k}\right)=\overset{p-1}{\underset{k=0}{\sum }}[f{\left(\Delta \Xi \right)}_{q}]2^{k},\\ =\sum_{k=0}^{p-1}f\;\left[\;\left(\frac{g_{p}}{\sqrt{\delta }}\;\sqrt{\left|-2\;\left(\frac{{\left(\;g_{p}\right)}^{1-q}-1}{1-q}\;\right)\right|}\right)-\left(\frac{g_{c}}{\sqrt{\delta }}\;\sqrt{\left|-2\left(\frac{{\left(\;g_{c}\right)}^{1-q}-1}{1-q}\;\right)\right|}\;\right)\;\right]2^{k},\\ =\sum_{k=0}^{p-1}f\;\left[\;\left(\;g_{p}\sqrt{\left|-2\;\left(\frac{{\left(\;g_{p}\right)}^{1-q}-1}{1-q}\;\right)\right|}\right)-\left(\;g_{c}\sqrt{\left|-2\left(\frac{{\left(\;g_{c}\right)}^{1-q}-1}{1-q}\;\right)\right|}\;\right)\;\right]2^{k}, \end{array}$$

The proposed QELBP has the ability to capture the image’s small changes of gray values, which represents the image textures as low-frequency components in gray values. The proposed model divides the input image (I) into non-overlapping blocks (B) with size of 3 × 3 of total (n) blocks. Then the proposed QELBP is extracted for each block (i) as defined by Equation (9). The algorithmic steps for obtaining the QELBP are presented in Algorithm 1. The Matlab code can be shared upon request by the authors.
**Algorithm 1:QELBP**1:Initialization: *I* = Input image, set the quantum parameter q = 0.62:**For** each Input image I do3:[*B*_1_*,* *B*_2*, …,*_ *B_n_*] ← I// Divide I into non-overlapped blocks size of *3* × *3* pixels4:**For***I* = 1 to *n* do5:$QELBP_{i}\;\leftarrow \;$*I*//Calculate QELBP for each block (i) as defined in Equation (9), where *i* denotes6:The *i*th block of 3 × 3 dimension.7:**End** For8:QELBP ←  I = [1,2,…n]// Final QELBP Features of all (n) blocks9:**End** For

### 3.4. CNN Architecture for Feature Extraction

**Figure 2:**
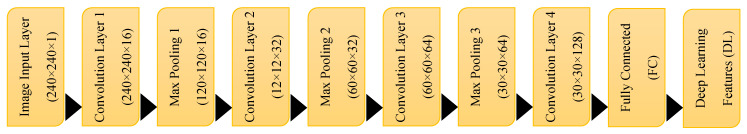
Structure of DL feature extraction.

CNNs have been lately applied in a variety of applications because of their capabilities for feature representation, patterns detection and classification. The main architecture of CNNs includes two essential parts; a feature extractor and a classifier. The feature extractor consists of several connected layers in sequence [18]. The CNNs consist of several convolution layers (Conv) and pooling layers, activation function, dropout and fully connected layer. The layers of CNNs are employed to transform the MRI scan into the chosen output after training [8]. Each layer comprises a set of small parameterized filters, named kernels which are applied independently to every layer. How far the kernel filter convolves around the input volume by shifting from one position to another is called stride. The output volume shrinks as the stride increases. Because of striding, the spatial dimensions of the output volume decrease significantly after every layer, and this impacts the performance of CNNs. The activation layer with ReLU activation function, is used to eliminate the negative numbers in the feature maps [19]. Then the rectified features are passed over pooling layers, also named down sampling layer. Two common functions that are frequently used in the pooling layer of CNNs, are the max and average pooling functions. The max pooling is used in this study to determine the maximum number in every sub-region. The feature maps are normalized by using a batch normalization layer that is used as a regulator for the CNNs’ training process. Generally, the gradient-based optimization algorithm is used to decrease an error function of CNNs to produce an extremely improved weight. The structure of proposed DL is shown in Figure 2.

### 3.5. LSTM Classifier

The LSTM was developed by Hochreiter and Schmidhuber to deal with the limitation of ANN in sequential data problems. It could be considered a special type of the recurrent neural network, which is capable of learning dependencies for prolonged periods and remember important information from previous processing steps. LSTM has been used in different tasks such in natural language processing [20], speech recognition [21] and can be appropriate to the MRI classifier. The LSTM is used for sequential data or time series data, but it can also be used for classification due to its ability to recognize images features across time by the connected memory blocks through its layers. The time series in the mathematical expression of LSTM indicates the length of the input sequence. In this study, the time series is represented by proposing QELBP–DL feature vector. LSTM has four interacting layers that are formed as a chain structure [22]. The classic LSTM normally has memory cells. In this study, the LSTM network includes 7 layers; sequence input with 12 dimensions that comes from combined extracted features of each MRI brain scan, 200-hidden units and 20% drop out. Additionally, the LSTM network was trained by using the Adam optimization method, where the maximum epoch value was set to 500 and the gradient threshold value was set to 1.

### 3.6. Evaluation Metrics

Five metrics are used here to evaluate the proposed model: accuracy (ACC), sensitivity (SENS), Specificity (SPEC), Precision (PRES) and area under the receiver operating curve (AUC):

(10) $$\begin{array}{c} \mathrm{ACC}=\frac{\mathrm{TP}+\mathrm{TN}}{\mathrm{TP}+\mathrm{FP}+\mathrm{TN}+\mathrm{FN}},\\ \mathrm{SENS}=\frac{\mathrm{TP}}{\mathrm{TP}+\mathrm{FN}},\\ \mathrm{SPEC}=\frac{\mathrm{TN}}{\mathrm{TN}+\mathrm{FP}},\\ \mathrm{PRES}=\frac{\mathrm{TP}}{\mathrm{TP}+\mathrm{FP}}, \end{array}$$

where TP, TN, FN and FP are demonstrated in Table 1.

## 4. Experimental Results

The experiments are carried out to show the effectiveness of proposed QELBP–DL as a feature extraction. The proposed model is tested on T2-w MRI images which are preferred modality to show the pathologic conditions for the clinicians to analyze the brain tumors. Seventy percent of the collected MRI brain scans were used in the training phase of the CNN and LSTM networks, while the remaining 30% were used for testing as unseen data.

**Figure 3:**
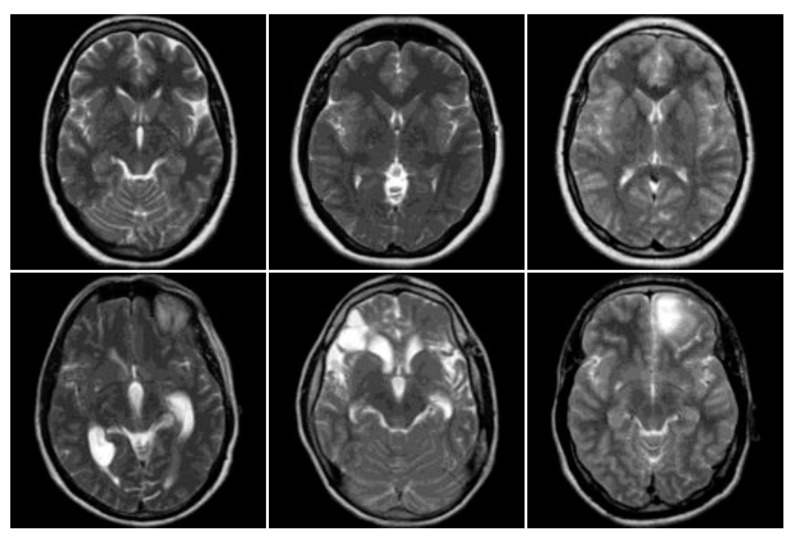
MRI brain images from the collected dataset.

Figure 3 shows a sample of the MRI images of normal and abnormal brain images from the composed dataset. The first row shows the MRI brain images that belong to healthy patients while the second row shows MRI brain images that belong to diseased patients.

**Figure 4:**
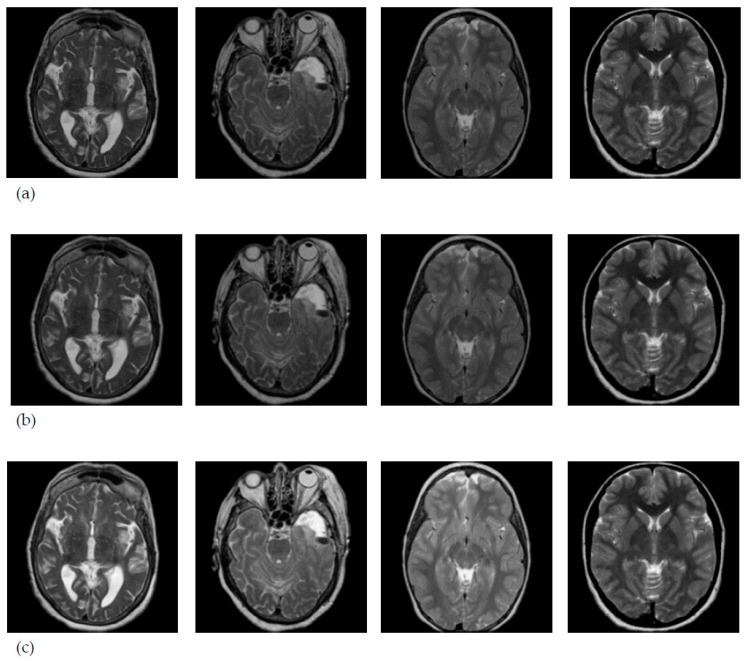
MRI brain images from the collected dataset (**a**) Original MRI images; (**b**) enhanced by the Gaussian filter with kernel of (3 × 3); (**c**) normalized MRI.

The MRI scans were enhanced by the Gaussian filter with kernel of (3 × 3) and normalized by histogram normalization, as shown in Figure 4.

The image enhancement as a preprocessing stage, was used to reduce the effects of random fluctuations in the intensity distribution of MRI scans which may affect the diagnosing process.

Although, the MRI considered as an efficient tool for the diagnosis of brain diseases, but the quality of MRI images suffering from image intensity variations due to the MRI scanners. Thus, the extracted results have different measures between the repeated scans or between different anatomic regions [23]. In addition, acquiring MRI data from different scanners at different sites produces variance in the dynamic intensity range of the brain tissue even though they are used the same acquisition protocol.

**Figure 5:**
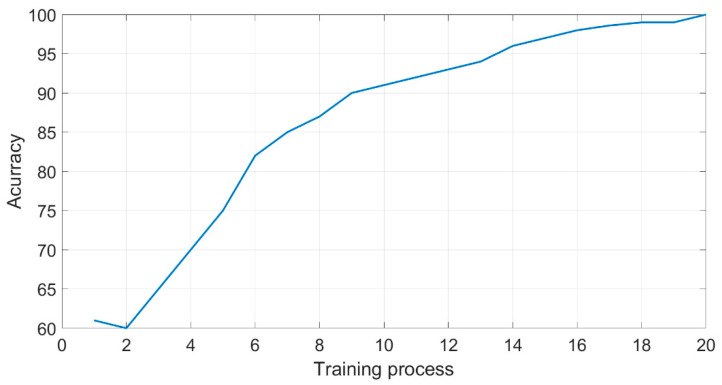
Training process.

The proposed CNN consists of nine layers in order to minimize the over-fitting by reducing the CNN architecture complexity. To preserve the complexity of all layers, the stride of 2 was used for all subsequent conventional layers which is equal to the original stride of the pooling layer to reduce the complexity of all conventional layers. The hyper-parameters of the CNN network were fixed to enable the convergence of the loss function in the training process. The training model (Figure 5) starts with a learning rate of 0.001 with the Adam Optimizer for 20 epochs using the categorical cross-entropy loss and validation frequency of 30 (the network is validated about once per epoch). The experiments are applied to Intel i7-6700HQ CPU (2.6 GHz) 8 GB RAM, 64-bit Windows 10, NVIDIA GTX 950 GPU, Matlab 2019b. The proposed CNN model was trained using 240 × 240 pixels input MRI. Reducing the training image size will help in reducing the complexity and the processing time of proposed CNN. After successful training, the test images (with a size of 512 × 512 pixels) were fed into the proposed trained model to obtain classification results.

### 4.1. Performance Evaluation of MRI Brain Classification

To test the effectiveness of the proposed method, we consider our collected datasets of brain MRI images. The proposed study consists of two features extraction stages, namely, QELBP by local binary patterns (LBP) of quantum entropy (QE) and DL by deep neural network. We have implemented three experiments to show the effectiveness of proposed QELBP–DL as a feature extraction as illustrated in Table 2. The aim of proposed (QELBP–DL) feature extraction method is to improve the accuracy of brain tumor classification in MRI images. For validating the classification results given by the proposed QELBP–DL approach, we consider the associating the true negative (TN), true positive (TP) and area under the curve (AUC).

When we look at the results reported in Table 2, the proposed method QELBP–DL achieves the best accuracy compared to the QELBP and DL feature extraction. This shows that the proposed combined features extraction method (QELBP–DL) performs better regardless of image contents.

The key steps of the proposed QELBP–DL feature extraction method are QELBP and DL. Furthermore, three pre-trained deep learning networks (AlexNet [24], GoogleNet [25] and SqueezeNet [26]) were used to extract features from our collected brain MRI scans dataset and compare their performances with the proposed QELBP–DL as presented in Table 3. Accordingly, AlexNet includes eight layers and 1000 classes, GoogleNet includes 144 layers and 1000 classes and the SqueezeNet includes 1000 classes. When we compare the results of existing methods with the proposed QELBP–DL method, the proposed method is better than the existing methods in terms of the average classification rate.

### 4.2. Comparative Analysis for of MRI Brain Classification

To show the effectiveness of proposed study, we compared it with previous studies reported in years (2016–2020), which used different brain MRI scans datasets is shown in Table 4. The motivation of chosen the above methods for comparative study is that, the primary objective of the existing methods is the classification of brain tumors as normal or abnormal from MRI scans using different approaches with different MRI datasets. Anitha and Murugavalli, 2016 [27], proposed the DWT with self-organizing map as a feature extraction and KNN as the classifier with custom brain MRI scans dataset of brain MRI. The accuracy achieved was 96.6% evaluated with only 55 brain MRI. Sachdeva et al. 2016 [7], applied the PCA with ANN with custom brain MRI scans datasets collected from Institute of Medical Education and Research (PGIMER), Chandigarh, India. The obtained accuracy was 91%. Their experiments were performed using a large number of features for MR brain tumor slices. Sultan, H et al. 2019 [28], proposed a new CNN model with 16 layers using custom brain MRI scans dataset collected from Tianjing Medical University, China. This study achieved best overall accuracy of 96.13% on T1-weighted contrast-enhanced images without using fold cross-validation. Badža M. et al. 2020 [29], proposed a new 22 layers CNN, using custom brain MRI scans dataset collected from Tianjing Medical University, China as well. This approach achieved an accuracy of 96.56% for classification of three tumor types tested on T1-weighted contrast-enhanced MRI. The performance of the proposed network was evaluated using 10-fold cross-validation. The above mentioned approaches achieved a lower classification rate than the proposed method. Finally, Raja et al. 2020 [30], proposed the hybrid approach of deep auto-encoder combined and Bayesian clustering, applied on public standard BRATS 2015 brain MRI database. This method is better than the other existing methods in classification accuracy, which is approaching the accuracy of the proposed method, but showing less accuracy, sensitivity and precision values compared with the proposed method. In summary, for different data sets with different complexities, the proposed method reached the best accuracy compared to the previous studies. This shows that the proposed method works well for the classification of brain tumors from MRI scans. These results show that our network has a good generalization capability to be used as a support tool for radiologists in brain MRI diagnostics. In this study, the developmental model for extracting features by both QELBP and DL and combining them into a single feature set QELBP–DL represent the main contribution. The proposed QELBP is used as an image texture descriptor to capture the low-frequency components of pixel gray values with the deep features of the proposed CNN model have significantly improved the overall classification effectiveness of MRI brain images.

## 5. Conclusions

In this study, we have proposed a novel method for brain MRI scan classification based QELBP and the DL features. In extract the main texture features from brain MRI scan, we have proposed a novel QELBP model involving quantum entropy LBP combined with the proposed deep features which extracted the high level spatial features from MRI brain scans. This model integrates property of quantum calculus, which uses the natural logarithmic function to solve the nonlinear complexity of the spatial relationship of image pixels and the deep learning feature extraction.

Experimental results on two different datasets, show that the proposed QELBP–DL model outperforms the existing brain classification methods. This study has demonstrated that proposed QELBP–DL model could effectively improve the performance of MRI brain scans classification, significantly better than the existing methods. Even though the improvement may not pose as a breakthrough in the field, it is one step closer in that direction. Using larger datasets of public brain MRI images, datasets will be considered as the future work.

## Figures and Tables

**Table 1:** Elements of the output confusion matrix.

Actual Class	Predicted Class	
	Abnormal	Normal
Abnormal (positive)	TP	FN
Normal (negative)	FP	TN

**Table 2:** Comparisons of QELBP, DL and the proposed C QELBP–DL methods, respectively using LSTM with the collected dataset.

Methods	Accuracy 100%	TP 100%	TN 100%	AUC
QELBP	89.50	94	85	0.8489
DL	93.50	98	89	0.9259
Proposed QELBP–DL	98.80	99	97.80	0.9864

**Table 3:** Comparisons of the proposed QELBP–DL proposed method with other pre-trained networks using the collected brain MRI scans dataset.

Method	Accuracy 100%	Features Dimensions	TP 100%	TN 100%
AlexNet [24]	92	4096	92	86
GoogleNet [25]	90	1000	96	83
SqueezeNet [26]	94	1000	97	88
Proposed QELBP–DL	98.80	12	99	97.80

**Table 4:** Comparisons of the proposed model with other methods using different brain MRI scans datasets.

Methods	Dataset Used	Accuracy 100%	Sensitivity 100%	Specificity 100%	Precision 100%	TP 100%	TN 100%
Anitha and Murugavalli, 2016 [27]	Custom dataset-2	96.60	88	33	96	96	100
Sachdeva et al. 2016 [7]	Institute of Medical Education and Research, Chandigarh, India	91	x	x	x	x	x
Sultan, H et al. 2019 [28]	Tianjing Medical University, China	96.13	93	97	95	93	97
Badža M et al. 2020 [29]	Tianjing Medical University, China	96.56	97	96	94	96	95
Raja et al. 2020 [30]	BRATS 2015 database	98.50	96	99	96	98	96
Proposed	Custom datasets	98.80	98	99	97	99	97.8

## References

[1] Rosenberg R.N. (2019). Atlas of Clinical Neurology.

[2] Işın A., Direkoğlu C., Şah M. (2016). Review of MRI-based brain tumor image segmentation using deep learning methods. Procedia Comput. Sci..

[3] Jalab H.A., Hasan A. (2019). Magnetic resonance imaging segmentation techniques of brain tumors: A review. Arch. Neurosci..

[4] Hasan A.M., Meziane F., Jalab H.A. Performance of grey level statistic features versus Gabor wavelet for screening MRI brain tumors: A comparative study. Proceedings of the 2016 6th International Conference on Information Communication and Management (ICICM).

[5] Nabizadeh N., Kubat M. (2015). Brain tumors detection and segmentation in MR images: Gabor wavelet vs. statistical features. Comput. Electr. Eng..

[6] Liang H., Li Q. (2016). Hyperspectral imagery classification using sparse representations of convolutional neural network features. Remote Sens..

[7] Sachdeva J., Kumar V., Gupta I., Khandelwal N., Ahuja C.K. (2016). A package-SFERCB-“Segmentation, feature extraction, reduction and classification analysis by both SVM and ANN for brain tumors”. Appl. Soft Comput..

[8] Hasan A.M., Jalab H.A., Meziane F., Kahtan H., Al-Ahmad A.S. (2019). Combining deep and handcrafted image features for MRI brain scan classification. IEEE Access.

[9] Yang X., Fan Y. Feature extraction using convolutional neural networks for multi-atlas based image segmentation. Proceedings of the Medical Imaging 2018: Image Processing.

[10] Chen Y., Jiang H., Li C., Jia X., Ghamisi P. (2016). Deep feature extraction and classification of hyperspectral images based on convolutional neural networks. IEEE Trans. Geosci. Remote Sens..

[11] Lai Z., Deng H. (2018). Medical Image Classification Based on Deep Features Extracted by Deep Model and Statistic Feature Fusion with Multilayer Perceptron. Comput. Intell. Neurosci..

[12] Despotović I., Goossens B., Philips W. (2015). MRI segmentation of the human brain: Challenges, methods, and applications. Comput. Math. Methods Med..

[13] Hasan A.M. (2017). An Automated System for the Classification and Segmentation of Brain Tumours in MRI Images based on the Modified Grey Level Co-Occurrence Matrix. Ph.D. Thesis.

[14] Hasan A.M., Meziane F., Aspin R., Jalab H.A. MRI brain scan classification using novel 3-D statistical features. Proceedings of the Second International Conference on Internet of things, Data and Cloud Computing.

[15] Toudjeu I.T., Tapamo J.-R. (2019). Circular Derivative Local Binary Pattern Feature Description for Facial Expression Recognition. Adv. Electr. Comput. Eng..

[16] Umarov S., Tsallis C., Steinberg S. (2008). On a q-central limit theorem consistent with nonextensive statistical mechanics. Milan J. Math..

[17] Marsaglia G., Bray T.A. (1964). A convenient method for generating normal variables. SIAM Rev..

[18] Alom M.Z., Taha T.M., Yakopcic C., Westberg S., Sidike P., Nasrin M.S., Hasan M., Van Essen B.C., Awwal A.A., Asari V.K. (2019). A state-of-the-art survey on deep learning theory and architectures. Electronics.

[19] Lundervold A.S., Lundervold A. (2019). An overview of deep learning in medical imaging focusing on MRI. Zeitschrift für Medizinische Physik.

[20] Palangi H., Deng L., Shen Y., Gao J., He X., Chen J., Song X., Ward R. (2016). Deep sentence embedding using long short-term memory networks: Analysis and application to information retrieval. IEEE/ACM Trans. Audio Speech Lang. Process..

[21] Sak H., Senior A.W., Beaufays F. Long short-term memory recurrent neural network architectures for large scale acoustic modeling. Proceedings of the 15th Annual Conference of the International Speech Communication Association(INTERSPEECH 2014).

[22] Le X.-H., Ho H.V., Lee G., Jung S. (2019). Application of long short-term memory (LSTM) neural network for flood forecasting. Water.

[23] Loizou C.P., Pantziaris M., Seimenis I., Pattichis C.S. Brain MR image normalization in texture analysis of multiple sclerosis. Proceedings of the 2009 9th International Conference on Information Technology and Applications in Biomedicine.

[24] Russakovsky O., Deng J., Su H., Krause J., Satheesh S., Ma S., Huang Z., Karpathy A., Khosla A., Bernstein M. (2015). Imagenet large scale visual recognition challenge. Int. J. Comput. Vis..

[25] Zhou B., Lapedriza A., Torralba A., Oliva A. (2017). Places: An image database for deep scene understanding. J. Vis..

[26] 26. Iandola F.N., Han S., Moskewicz M.W., Ashraf K., Dally W.J., Keutzer K. (2017). SqueezeNet: AlexNet-level accuracy with 50x fewer parameters and <0.5 MB model size. arXiv.

[27] Anitha V., Murugavalli S. (2016). Brain tumour classification using two-tier classifier with adaptive segmentation technique. IET Comput. Vis..

[28] Sultan H.H., Salem N.M., Al-Atabany W. (2019). Multi-classification of brain tumor images using deep neural network. IEEE Access.

[29] Badža M.M., Barjaktarović M.Č. (2020). Classification of Brain Tumors from MRI Images Using a Convolutional Neural Network. Appl. Sci..

[30] Raja P.S. (2020). Brain tumor classification using a hybrid deep autoencoder with Bayesian fuzzy clustering-based segmentation approach. Biocybern. Biomed. Eng..

